# Mental health care practitioners’ understanding of the policy guideline on 72-hour assessment

**DOI:** 10.4102/curationis.v48i1.2660

**Published:** 2025-04-21

**Authors:** Ontlotlile I. Mpheng, Leepile A. Sehularo, Miriam M. Moagi, Gaotswake P. Kovane

**Affiliations:** 1 NuMIQ Research Focus Area, Faculty of Health Sciences, North-West University, Potchefstroom, South Africa; 2 Lifestyle Diseases Research Focus Area, Faculty of Health Sciences, North-West University, Mahikeng, South Africa; 3 Department of Nursing, Faculty of Health Sciences, University of Limpopo, Sovenga, South Africa

**Keywords:** 72-hour assessment, 72-hour policy guidelines, mental health, mental healthcare practitioners, mental healthcare users

## Abstract

**Background:**

The policy guidelines on 72-hour assessment of involuntary Mental Health Care Users (MHCUs) are implemented in respect of involuntary MHCUs who need mental health care, treatment and rehabilitation services. Currently, there is poor implementation of the policy guidelines on 72-hour assessment of involuntary MHCUs. This includes MHCUs staying beyond 72-hour assessment period and being exposed to infringement and violation of their rights.

**Objectives:**

This study explored and described the Mental Health Care Practitioners’ (MHCPs) understanding of the current practice of the policy guidelines on 72-hour assessment of involuntary MHCUs in South Africa.

**Method:**

A qualitative, exploratory, descriptive research design was followed. Participants comprised of MHCPs and were purposively sampled, while data were gathered through Focus Group Discussions utilising Microsoft Teams. Data analysis employed the six steps of thematic analysis to assess data, generate themes and categories presented concurrently with MHCPs direct quotations.

**Results:**

Three themes emerged after data analysis namely, (1) MHCPs’ understanding of the policy guidelines on 72-hour assessment of involuntary MHCUs; (2) MHCPs’ challenges with the policy guidelines on 72-hour assessment of involuntary MHCUs; and (3) MHCPs’ suggestions to strengthen the policy guidelines on 72-hour assessment of involuntary MHCUs.

**Conclusion:**

The findings of the study indicated that there should be well-trained administrators, qualified MHCPs, appropriate infrastructure, and community and family involvement to ensure proper implementation of the 72-hour policy guidelines.

**Contribution:**

This study highlights that with the proper implementation of the 72-hour policy guidelines, MHCU rights can be protected, therefore contributing to proper mental illness management.

## Introduction

The Policy Guidelines on 72-hour assessment of involuntary Mental Health Care Users (MHCUs) are implemented for MHCUs who need treatment, care and rehabilitation. However, these MHCUs lack the competence to consent for their mental health treatment and rehabilitation services (Narsi 2022). These involuntary MHCUs are harmful to themselves and others, because of their mental breakdown and their poor insight (Act No. 17 of 2002). According to the *Mental Health Care Act* (MHCA) (Act No. 17 of 2002), the Policy Guidelines on 72-hour assessment of involuntary MHCUs recommend and provide protocols on emergency mental health management. The policy guidelines are established under Section 21 of the *National Health Act* and are intended to provide guidance to health authorities and healthcare practitioners on the prerequisites for clearance to conduct 72-hour assessments. The policy guidelines detail the processes for conducting these assessments on individuals receiving involuntary mental healthcare.

Rendering of 72-hour assessment of involuntary MHCUs is vital for observation for ruling out medical conditions, before referral to a mental healthcare institution where further care, treatment and rehabilitation services are provided (Marufu 2019; Narsi 2022). Diverse mental healthcare practitioners (MHCPs) are required for provision of involuntary mental health treatment. Mental Health Care Practitioner refers to a registered medical practitioner, psychiatrist, nurse, psychologist, occupational therapist or social worker who has been trained to provide the prescribed mental healthcare, treatment and rehabilitation services to patients who so require such services (MHCA [Act No. 17 of 2002]). These MHCPs implement mental health legislations such as the MHCA (Act No. 17 of 2002) and Policy Guidelines on 72-hour assessment of involuntary MHCUs when they provide care to MHCUs. While on the one hand, there are concerns with regard to the application of policy guidelines in terms of the 72-hour assessment of involuntary mental health units (Brathovde 2021; Morris 2021), on the other hand, newly practising MHCPs report a lack of confidence regarding the management of MHCUs who are admitted under 72-hours assessment (Goldsmith et al. 2021). This was supported by Mamabolo (2021) and Narsi (2022) who indicated in their findings that some MHCPs do not fully understand the procedures provided under the policy guidelines of 72-hour assessment, which makes care, treatment and rehabilitation of MHCUs admitted under 72-hour admission reprehensible, verging on the unethical (Mamabolo 2021; Narsi 2022).

Healthcare professionals point to a lack of human resources when providing mental health services to MHCUs that are admitted for a 72-hour period (Narsi 2022). Mamabolo (2021) concurs that a lack of human resources has a detrimental impact regarding provision of 72-hour assessment of involuntary MHCUs. In addition, Narsi (2022) shares that healthcare professionals also report that infrastructure is insufficient to support MHCUs during the 72-hour admission. There are compounding problems such as the high turnover rate of MHCUs in the observation wards and occasions when MHCUs stay longer than initially anticipated (Maila, Martin & Chipps 2020). Furthermore, research indicates that the policy requirements for the 72-hour assessment of involuntary mental health units are not implemented professionally (Morris 2021). The MHCPs report reduced levels of compassion satisfaction and burnout as a result of working excessive hours (Mthombeni 2021; Mufti & Zirinsky 2021). Moreover, lack of human resources is perceived as a contributory factor to failure to implement mental health treatment implementation during admission, assessment and care under the 72-hour admission (Morris 2021; Narsi 2022). The researcher noted that misdiagnosing of MHCUs, incorrect admission procedures and inappropriate admissions to designated 72-hour health facilities, may be the reason emanating from improper implementation of the policy guidelines on 72-hour assessment of involuntary MHCUs. In addition, researchers argue that the insufficient number of skilled personnel, limited access to mental health services and a lack of understanding of the MHCA forms contribute to inadequate care, treatment and rehabilitation for involuntary MHCUs. Because of the aforementioned challenges, suggestions for enhancing the implementation of the policy guidelines on the 72-hour assessment of involuntary assessment facilities are proffered in the penultimate section of this article.

## Research methods and design

### Study design

This article is part of a PhD study, which followed a qualitative, exploratory, descriptive and contextual research design (Polit & Beck 2017). Researcher explored and described the study phenomenon in the literature through employing a qualitative, exploratory, descriptive and contextual research methodology (Ahmad & Aini 2021). This research design seeks to explore the issues under investigation from the perspective of the participants. This research design allowed the researchers to collect in-depth information for the development of a Practice Model (PM) to strengthen the implementation of policy guidelines on 72-hour assessment of involuntary MHCUs (Grove, Burns & Gray 2013).

### Study setting

The study was conducted at general hospitals designated for 72-hour assessment of involuntary MHCUs in three provinces of South Africa: North West province (NWP), Gauteng province (GP), and Northern Cape province (NCP). The general hospitals have 72-hour health facilities or units for admission of involuntary MHCUs for 72-hour assessment. Three Focus Group Discussions (FGDs), with one from each province was conducted from each hospital. Regardless of the challenges encountered in all nine South African provinces, the three provinces were chosen because there is a significant challenge of conducting policy guidance for 72-hour assessments of involuntary MHCUs in these provinces (Docrat et al. 2019). The authors further shared indication of high influx of admissions among these provinces (NCP, 98.7%; NWP, 96.9% and GP, 95.1%).

### Study population and sampling strategy

The population consisted of MHCPs working in designated 72-hour general health facility for conducting 72-hour assessment for involuntary MHCUs. Participants with minimum of 1 year experience of caring for involuntary MHCUs were included in the study. The MHCPs were selected through quota sampling, with each province representing a specific quota (Creswell 2014:79–80). The rationale behind using the quota sampling method in this study was to ensure the inclusion of participants who shared similar characteristics, such as work experience of more than 3 years and profession (Bhardwaj 2019:162). In addition, purposive sampling was employed within each quota to specifically target MHCPs who were most knowledgeable about the study’s research question (Polit & Beck 2021).

The recruitment process was facilitated by mediators who were facility managers from each hospital as they know experienced and required participants as per the recruitment material shared. Following the facility managers’ identification of potential participants, the researcher gave them a brief overview of the study through WhatsApp or call considering the participants preference. Potential participants were informed about consent forms being facilitated by an independent person who is a mental health specialist working as a senior lecturer at the North-West University (NWU), for purpose of maintaining ethical standards. Explaining of consent forms was then carried out through an online Microsoft Teams meeting. Potential participants were given a minimum of 24-hours to respond back regarding signing of consent forms. Participants who eventually participated in the study signed consent forms.

### Data collection

Once approval was obtained from the Department of Health (DoH 2002) and permission was granted by the hospitals, the recruitment of participants was facilitated by mediators, specifically Operational Managers (OP). The researcher communicated with each mediator via email, WhatsApp, or phone call to coordinate the recruitment process. Subsequently, FGDs were organised, bringing together the researcher and participants from each province. Three FGDs were conducted, one FGD per province, through Microsoft Teams to gather data from the MHCPs from the three provinces (NCP-7, NWP-6 and GP-6), considering geographical distance between provinces. Before data collection, consent was obtained from MHCPs regarding the recording of the FGDs. Virtual semi-structured FGDs were employed, with the goal of providing researcher with a flexible technique (Polit & Beck 2017). Furthermore, virtual semi-structured FGDs were used to collect detailed information from research participants and that allowed the researcher as the interviewer to make follow-up questions. The FGD provided an advantage to the participants to stimulate each other’s thinking for more information. During the interviews, participants were asked to keep their cameras on, with the assurance that their anonymity would be preserved when the study’s findings were presented. To maintain confidentiality, no names were recorded or used during data collection or in the reporting of the results. The 19 MHCPs who participated in this study included 9 professional nurses, 1 social worker, 5 medical doctors and 4 clinical psychologists. The professional nurses as dominating participants are said to comprise 50% of the global healthcare workforce and contribute significantly to patient healthcare (Godsey, Houghton & Hayes 2020). The FGDs lasted between 30 min and 60 min; all the participants were fluent in communicating in English and researcher faced no challenges related to language barrier. The recorded FGDs were transcribed verbatim by the researcher immediately after data collection.

The interview scheduled questions were as follows:

- What is your understanding of the current practice regarding policy guidelines on 72-hour assessment of involuntary MHCUs?
- What is your understanding of the current practice regarding the implementation of policy guidelines on 72-hour assessment of involuntary MHCUs?
- What can be done to strengthen the implementation of policy guidelines on 72-hour assessment of involuntary MHCUs?

### Data analysis

Data saturation was reached on the third FGD. Braun and Clarke’s six steps of thematic analysis were used to assess data (Clarke & Braun 2017). Thematic analysis involves identifying recurring themes, topics, ideas, patterns, and meaningful categories that emerge from the data. The first step involved familiarisation with the data by going through all the data scripts individually, by the researcher, main supervisor and the independent coder to ensure dependability. This was followed by developing the emerging codes through highlighting and grouping similar codes, ensuing identification of themes. Different topics were examined, followed by the establishment of themes and compilation of a summary of the results. After undertaking separate data analyses, the independent coder, the researcher and the main supervisor convened using the Microsoft Teams platform to determine the ultimate themes and sub-themes. Participants’ personal information and data collected were kept confidential. All recordings and transcriptions were saved with a password and are kept in a locked cupboard. Only the researchers and the study’s independent coder will have access to the data.

### Trustworthiness in qualitative studies

The criteria for guaranteeing trustworthiness – transferability, confirmability, authenticity, credibility and dependability – were satisfied (Polit & Beck 2017). These crucial aspects of trustworthiness in qualitative research, collectively ensure the truthfulness of the findings. These elements help validate the accuracy of the research process and the integrity of its conclusions. After the data were transcribed, an independent coder was involved to confirm authenticity. A co-coder enabled the researcher to identify patterns and connections within the data, ensuring the accuracy of the findings and assessing how effective results are. The results of the study were thoroughly described after a thematic analysis of the data. Transferability was guaranteed by offering a comprehensive explanation of the study’s findings following data collection and analysis, through writing of manuscripts and a thesis. Transferability is a crucial criterion that measures the extent to which a -study’s findings can be applied to different contexts and settings. Confirmability was guaranteed by providing a thorough explanation of the research methodology. Credibility was preserved by interacting with research findings over time and grouping similar themes.

### Ethical considerations

Approval to conduct the study was granted by the School of Nursing Scientific Committee (NuMIQ) and the North-West University Health Research Ethics Committee (NWU-HREC- 00032-23-A1). The study was also approved by the North West Provincial Department of Health (DoH), the Gauteng Provincial DOH and the Northern Cape Provincial DoH, as well as a goodwill permission letter from the Head of Health Establishment (HHE) of the hospitals at which data were collected. All study participants provided informed consent and ethical principles, namely autonomy, beneficence, justice and non-maleficence, were adhered to in this study.

## Results

### Demographic profile of the participants

In total, 19 participants participated in three FGDs. The 19 MHCPs (3 males and 16 females) who participated in this study included 9 professional nurses, one social worker, 5 medical doctors, and 4 clinical psychologists. The MHCPs age ranged between 29-59 years. Table 1 below provides the demographic profile of participants.

**TABLE 1:** Demographic profile of participants.

Demographic details of participants			
Participant	Age in years	Occupation in MHCPs	Gender
**FGD 1 – NCP MHCPs participants**			
Participant A	32	Medical Doctor	M
Participant B	32	Professional Nurse	F
Participant C	52	Professional Nurse	F
Participant D	33	Social Worker	F
Participant E	36	Professional Nurse	F
Participant F	29	Professional Nurse	F
Participant G	53	Medical Doctor	M
**FGD 2 – NWP MHCPs participants**			
Participant A	46	Professional Nurse	F
Participant B	46	Professional Nurse	F
Participant C	40	Clinical Psychologist	F
Participant D	46	Clinical Psychologist	F
Participant E	44	Medical Doctor	F
Participant F	54	Medical Doctor	M
**FGD 3 – GP MHCPs participants**			
Participant A	46	Professional Nurse	F
Participant B	53	Professional Nurse	F
Participant C	59	Professional Nurse	F
Participant D	37	Clinical Psychologist	F
Participant E	33	Clinical Psychologist	F
Participant F	29	Medical Doctor	F

Note: Total participants were 19 MHCPs.

MHCPs, Mental Healthcare Practitioners; NWP, North West province; NCP, Northern Cape province; GP, Gauteng province; FGD, Focus Group Discussions; F, female; M, male.

### Presentation of findings

#### Themes

Three FGDs were conducted with the MHCPs. Three themes and 29 subthemes emerged from the data after analysis as represented in Table 2. Each theme contains an outline and sub-themes, followed by direct quotes of participants.

**TABLE 2:** Themes and the related sub-themes.

Themes	Sub-themes
1. MHCPs understanding of the policy guideline on 72-hour assessment of involuntary MHCUs	1.1 Application procedure and forms 1.2 Assessment procedures and forms 1.3 Outcomes of 72-hour assessment 1.4 Head of health establishment procedures 1.5 72-hour assessment at a designated facility 1.6 72-hour assessment to exclude physical conditions 1.7 72-hour assessment in a safe environment 1.8 72-hour assessment by competent professionals 1.9 72-hour assessment when involuntary admission is indicated 1.10 Legal protection of MHCUs
2. MHCPs challenges with the policy guideline on 72-hour assessment of involuntary MHCUs	2.1 Inadequate access to psychiatric hospitals 2.2 Ineffective completion of forms 2.3 Challenges in application and interpretation of the guidelines 2.4 MHCU-related challenges 2.5 Family-related challenges 2.6 Inadequate 72-hour assessment facilities 2.7 Lack of 72-hour assessment facilities 2.8 Human resources’ attitude challenges 2.9 Insufficient human resources 2.10 Other resource-related challenges 2.11 Medical-legal risks 2.12 Patient care challenges 2.13 Overcrowding in 72-hour assessment facilities 2.14 Readmissions and its consequences
3. MHCPs suggestions to strengthen the policy guideline on 72-hour assessment of involuntary MHCUs	3.1 Adequate infrastructure and resources 3.2 Accessible services (assessment, treatment and/or rehabilitation) 3.3 Sufficient human resources 3.4 Competent human resources 3.5 Governance of guidelines 3.6 Community and stakeholder empowerment 3.7 Family empowerment

MHCUs, Mental Health Care Users; MHCPs, Mental Health Care Practitioners.

#### Theme 1: Mental Health Care Practitioners’ understanding of the policy guideline on 72-hour assessment of involuntary Mental Health Care Users

The sub-themes that emerged from the MHCPs’ understanding of the policy guidelines on 72-hour assessment of involuntary MHCUs are elaborated hereunder:

**Sub-theme 1.1: Application procedure and forms:** The MHCPs understand that there are procedures and different forms that must be completed prior to or on admission and during provisioning of care, treatment and rehabilitation of the MHCUs, as stipulated in the 72-hour policy guideline. The procedures require the presence or availability of a family member, a medical doctor or psychiatrist, a professional nurse and at times a clinical psychologist and a social worker:

‘Yes, according to my understanding … the policy guidelines regarding … involuntary admission of mental health care users is that … we need the application that is from the applicant, that is Form 4 that can be rendered by a healthcare professional. If there’s no family member or a guardian or a neighbour or a family member … A patient has to have that Form 4.’ (GP, FGD2, Participant D)

‘Depending on who brings the patient or who brings the individual if it is the police we should be expecting a form 22 and should be signed by the police.’ (NCP, FGD1, Participant D)

‘What I understand is that when patient is admitted as involuntary mental health care users, they should be admitted to a designated hospital with relevant mental health care forms, which is O4, O5 and O6.’ (NWP, FGD3, Participant B)

**Sub-theme 1.2: Assessment procedures and forms:** The MHCPs understand that there are assessment procedure and forms required when admitting the involuntary MHCUs for 72-hour assessment. According to the MHCPs understanding, the assessment of the MHCUs must be undertaken by two MHCPs, with involvement of a family member or spouse and the two written findings are then submitted to the HHE. Before admission, the HHE decides if the MHCU should be treated as an in-patient or out-patient and subsequently provides a notice to the applicant using Form MHCA 07. If the MHCU is admitted, they should stay in the facility for a maximum of 72-hours:

‘[*A*]nd then if the patient was brought by the family, [*patient*] history should be collected from the family side as collateral, including the patient themselves.’ (NCP, FGD1, Participant D)

‘It needs to be assessed by two medical officers, officer. They need to do a physical assessment and the psychological assessment.’ (GP, FGD2, Participant D)

‘The other reason for the guidelines was to avoid everyone being lumped, for the sake of being called mental health care users and being sent far away from home to a designated psychiatric hospital.’ (NWP, FGD3, Participant E)

**Sub-theme 1.3: Outcomes of 72-hour assessment:** Participants understand that according to the 72-hour policy guideline, the involuntary MHCU must stay for 72-hours in the 72-hour assessment unit. Within 24-hours prior the expiry of 72-hours assessment, the MHCPs record the findings and outcomes of 72-hours assessment in Form MHCA 06. If, according to assessment, the MHCU needs further admission, the MHCU is transferred to a mental health hospital or section of designated general hospital for delivery of psychiatric care through use of MHCA 11 and transitions from the HHE. The MHCPs confirmed their understanding regarding outcomes of the 72-hour assessment by the following quotations:

‘After ruling out medical condition, if the patient is still psychotic, the patient should be sent to a designated psychiatric hospital with relevant mental health care forms, which is O4, O5 and O6.’ (NWP, FGD3, Participant A)

‘[*O*]nly after only after 72-hour and if the patient has been declared mentally ill and need further treatment. Then there are other forms that, will be completed form 6 and form 8. For involuntary mental health care user.’ (GP, FGD2, Participant C)

‘[*A*]nd then taking it further, whether we are going to change our, what do you call say, whether are we still continue with involuntary that means the patient is still psychotic.’ (NCP, FGD1, Participant D)

**Sub-theme 1.4: Head of health establishment procedures:** The MHCPs understood the policy guideline about the role of the HHE as the one who decides if the MHCU must be treated as an in-patient or out-patient. Upon such decision, the HHE provides a notice to the applicant using Form MHCA 07 to the Mental Health Review Board. This finding is confirmed by the following quotations:

‘[*A*]nd once the patient has been successfully diagnosed and admitted through those forms, there is actually a form that should be filled by the health establishment and the individual. In most of the cases, [*this*] is the CEO of the hospital.’ (NCP, FGD1, Participant D)

‘[*A*]nd we need form 8 from the, from the health establishment again for us to continue and with the admissions in that case …’ (GP, FGD2, Participant D)

**Sub-theme 1.5: 72-hour assessment at a designated facility:** The MHCPs understand that the policy guideline stipulates that there must be designated facilities to provide 72-hour assessment of involuntary MHCUs:

‘I believe the policy guidelines they state that they have to be admitted in a facility that is mandated by the health, the mental Health Review Board, I presume. To actually perform these observation periods … it has to be in a facility that is mandated to do that where they can safely be observed during that 72-hour period.’ (GP, FGD2, Participant A)

‘So also, just make sure that where they are admitted eeh the structure is proper for such patients, especially those that are aggressive.’ (NWP, FGD3, Participant E)

‘… I think as pointed out that the facilities must be adequate to care for the mental? To care and treat mental health conditions as well as physical conditions.’ (NCP, FGD1, Participant B)

**Sub-theme 1.6: 72-hour assessment to exclude physical conditions:** Participants understand that the policy guidelines on 72-hour assessment of involuntary MHCUs is also aimed at excluding the medical conditions of the MHCU. According to the MHCPs, the 72-hour assessment period helps in ensuring that indeed the MHCUs has signs and symptoms related to mental illness:

‘What I understand is that when patients are admitted as involuntary mental health care users, they should be admitted to casualty to rule out any other medical conditions and to be admitted into a 72-hour observation ward or medical ward to rule out medical conditions.’ (NW, FGD3, Participant B)

‘Remember, patient needs to be we need to rule out medical conditions there so that we can assess if the patient has mental health problems …’ (GP, FGD2, Participant D)

‘The understanding of 72-hour assessment of the involuntary patient … there is assessment, the observation or the presentation of the patient? And based on whatever we’ll collect as information, we have to decide …, is it medical or psychological or psychiatric condition related? …’ (NCP, FGD1, Participant E)

**Sub-theme 1.7: 72-hour assessment in a safe environment:** The MHCPs understanding of the policy guideline is that it promotes safety of the MHCUs, those around them and their surroundings. The MHCPs further shared their understanding that by being in safe environment will ensure protection of the MHCUs, MHCPs, including other personnel:

‘We also need to ensure that the environment that they are in is safe from all those things that can be harmful to them … [*off*]. We should ensure that it is safe.’ (NWP, FGD3, Participant A)

‘[*B*]ecause our dilemma is always, is 1 security who stay far, away from the ward.’ (NCP, FGD1, Participant E)

‘I did not mean that, but anyway it has to be conducive for a mental health care users, free of hazards.’ (GP, FGD2, Participant D)

**Sub-theme 1.8: 72-hour assessment by competent professionals:** The participants shared their understanding that the 72-hour assessment of involuntary MHCUs needs to be performed by competent professionals who have undergone mental health training. During the 72-hour assessment the MHCPs should perform physical examination and competently do a mental status assessment of the involuntary MHCUs:

‘Also, those people that are working with those patients … the 72-hours patients may also need to be skilled in knowing how to handle them in case …’ (NWP, FGD3, Participant A)

‘[*B*]ecause now, remember that these patients when they come in most of the time and they are already psychotic, if we have the right individuals to take care of this patient, that will reduce the patient hospital stay … And if you’ve got relevant individuals in that that have interest or that have special skills.’ (NCP, FGD1, Participant D)

**Sub-theme 1.9: 72-hour assessment when involuntary admission is indicated:** The MHCPs shared their understanding that for a MHCU to meet admission criteria as an involuntary MHCU, the policy guidelines on 72-hour assessment of involuntary MHCPs, stipulate that the MHCPs should conduct a mental status examination or assessment of the patient (being violent, aggressive physically and verbally, a danger to themselves and those around them):

‘[*It’s*] not a question, but just realising that to meet the criteria for an involuntary admission, a patient has to have the mental illness that impairs their ability to accept care … so they’re actually refusing. OK, but they’re … they also have to harm and be a danger to either themselves or other people, including a danger to their reputation. I just wanted to add that.’ (GP, FGD2, Participant A)

‘According to my understanding uuh … for an involuntary admission that user can be admitted against their will, and this can only be done if the user, is experiencing a mental illness that is impairing their ability to make the best decision or to accept treatment, which could be helpful for them. So, the judgement and the reasoning or the insight into the condition will be impaired …’ (NWP, FGD3C, Participant B)

**Sub-theme 1.10: Legal protection of Mental Health Care Users:** The MHCPs expressed feelings of being overwhelmed, anxious and concerned about the legal protection of MHCUs. This concern stems from their understanding of the policy guidelines, which emphasise the legal protection of MHCUs. Their worries are particularly heightened by issues such as inadequate 72-hour assessment facilities, the incompetence of some MHCPs, shortcomings in the application and assessment procedures, and concerns about the safety of MHCUs – factors that seem to contradict the intentions of the 72-hour policy guidelines:

‘[*R*]emember the mental health by … they are covered by law, so we cannot just force even if we see this patient is a hazard to the society, but they are involuntarily coming here …’ (NCP, FGD1, Participant C)

‘The current but you know this one is supposed too it’s trying to protect the mental health care users so that we don’t mismanage them where everyone who comes with confusion will be regarded as mental illness.’ (NWP, FGD3, Participant F)

#### Theme 2: Mental Health Care Practitioners’ challenges with the policy guidelines on 72-hour assessment of involuntary Mental Health Care Users

The sub-themes that emerged from the challenges with the 72-hour policy guidelines on the assessment of involuntary MHCUs are outlined and discussed hereunder.

**Sub-theme 2.1: Inadequate access to psychiatric hospitals:** The participants shared a challenge regarding limited access of MHCUs to mental healthcare institutions because of psychiatric hospitals being invariably far from the MHCUs homes, and family members’ inability to visit the MHCUs because of compromised socio-economic status. The challenge lies in reconciling the policy guideline, which emphasises the importance of providing access to mental healthcare and ensuring that individuals with mental health conditions receive care closer to their homes:

‘There is a serious shortage of psychiatric hospitals in Gauteng Province, so sometimes we transfer patients to other provinces.’ (GP, FGD2, Participant C)

‘For me to some extent we are not really practising the policy because it talks about 72-hour observation, then we end up, we end up staying more than a week or two, and even a month because we’ll be waiting for a bed in there, in there the mental health institution 1, 2 then to some extent again we have a problem there the policy will … some patients will will patients will won’t be able to access the services of the mental institution.’ (NWP, FGD3, Participant F)

‘Because our patients are staying for a very long. Sometimes worse, the worst end they don’t take our patients. they will be forever seeing that they [*psychiatric hospital*] don’t have space so. Yeah, that is that.’ (NCP, FGD1, Participant C)

**Sub-theme 2.2: Ineffective completion of forms:** The MHCPs expressed a common challenge: some of them fail to complete the forms accurately, despite the requirement for assessments to be conducted in accordance with the 72-hour policy guidelines. They further indicated that this is evident as the forms mostly have similar information, as the MHCPs copy what is written from another MHCP, because of some ineptitude to make their initial assessment:

‘It’s a problem when filling the forms, especially because patients are from the district and there’s 04, it’s filled by the family. So, it comes from the District Hospital [*with*] wrong [*details*] and the family is not [*available for verification*].’ (NWP, FGD3, Participant B)

‘[*T*]he … shared that sometimes they seem to be confusion amongst colleagues in filling in the forms.’ (GP, FGD2, Participant A)

**Sub-theme 2.3: Challenges in application and interpretation of the guidelines:** The MHCPs agreed that there is a challenge regarding comprehensive understanding of the 72-hour policy guidelines and the procedures prescribed:

‘That’s what I’m saying. It’s a challenge for us. It’s difficult for us to implement that because … how can I give you a form that will be sent to the Review Board for 30 days whereas we are here for only three days? That’s why I’m saying it’s difficult for us to implement those policies … [*are unclear*] …’ (GP, FGD2, Participant D)

‘With understanding of the guideline. I’m really not sure if we understand the guideline because it’s saying involuntary, but we end up admitting assistant mental health care users for 72-hour, mental health observation … so we are not there’s lack of understanding into the guideline.’ (NWP, FGD3, Participant A)

**Sub-theme 2.4: Mental Health Care User-related challenges:** The participants shared that in most instances the MHCUs relapse and are readmitted because of not receiving services as soon as required, especially on issues of rehabilitation as most MHCUs are substance users. There are specific procedures that must be followed before an MHCU can receive timely mental health services, as outlined in the 72-hour assessment policy for involuntary MHCUs. However, in many cases, these services are not available:

‘We can increase the personnel as much as we want but … it’s a revolving door … they keep on coming back because once they go out to the community … those issues are not addressed.’ (NWP, FGD3, Participant E)

‘So what we have experienced is the very same people on 72-hours, especially the drug abuse. They still being supplied very same thing in the hospital.’ (NCP, FGD1, Participant E)

**Sub-theme 2.5: Family-related challenges:** The MHCPs highlighted a challenge where many families are reluctant to take responsibility for MHCUs and often prefer that the MHCU stay in the hospital longer than the duration specified by the guidelines:

‘Maybe … maybe the family member might be refusing for the relative to go for further care or to be admitted for psychiatric care. And the team might feel quite strongly that this mental health care user is a danger to themselves. They’re a danger to the community. We need [*them*] to be in the hospital.’ (GP, FGD 2, Participant A)

‘So now we are having that challenge that families, because they are ill informed, you know, they expect that because they brought a person to the hospital, therefore he must be kept for such and such a period …’ (NWP, FGD3, Participant E)

‘Maybe the family member might be refusing for the relative to go for further care or to be admitted for psychiatric care. And the team might feel quite strongly that this mental health care user is a danger to themselves …’ (GP, FGD1, Participant A)

**Sub-theme 2.6: Inadequate 72-hour assessment facilities:** The MHCPs raised a challenge regarding admission of involuntary MHCUs in hospitals that are not designated to provide 72-hour observation of involuntary MHCUs:

‘The infrastructure is not conducive. The guidelines says we should not mix adolescents, the minors with the elders, the elders above 60 and the juveniles and the youth, bo [*about*] 17 years, but because we don’t have … compatible infrastructure, we just mix them all and they end up being overcrowded. So, it’s very, very difficult to observe them thoroughly.’ (NWP, FGD3, Participant A)

‘Our challenge the implementation is suffering of the structural context where we are practising neh, that is obvious.’ (NCP, FGD1, Participant E)

‘So our female award is actually a mixed medical and mental health and medical ward. So some of the sisters may not be equipped to deal with Mental health patients there, that is one of the challenges that we have.’ (GP, FGD2, Participant F)

**Sub-theme 2.7: A lack of 72-hour assessment facilities:** There was a challenge across the provinces regarding lack of 72-hour assessment facilities. This lack of 72-hour assessment facilities is evident on admission of involuntary MHCUs far away from their homes, because in districts where they reside there are no 72-hour assessing facilities:

‘We are just taking care of patients, but we don’t have a 72-hour structure in the observation. When you go to the wards, you should have [*gone*] through the wards or are you going to go in there and walk around and see. It is terrible.’ (NCP, FGD1, Participant B)

‘And another thing having only … hospital as the listed 72-hour observation institution. It becomes a problem because now of over-crowding…’ (NWP, FGD3, Participant F)

**Sub-theme 2.8: Human resources’ attitude challenges:** The participants in this study identified a major obstacle to the effective implementation of the 72-hour assessment policy for involuntary MHCUs. This challenge is primarily attributed to the attitudes of human resources in the mental healthcare institutions where the participants work, particularly because of a lack of understanding regarding how to properly care for involuntary MHCUs:

‘It is unlawful because it doesn’t comply with the *Mental Health Care Act*. We are just on our own, as the sister said we are rural. So, we have to sort ourselves, because of non-compliance. If anything happens, we are on our own because we don’t comply to the *Mental Health Care Act*. We are just taking care of patients, but we don’t have a 72-hour structure in the observation.’ (NCP, FGD1, Participant B)

‘From the implementation from our side like we have already mentioned due to unforeseen other issues that we are actually facing, we are honestly unable to apply or implement, the norm as per the act, as a result most of the things that we do on or implement they’re actually unlawful as she has mentioned.’ (NWP. FGD3, Participant E)

**Sub-theme 2.9: Insufficient human resources:** The MHCPs revealed insufficient human resource challenge. They highlighted that this limitation poses a significant challenge when conducting the 72-hour assessment required for the care of involuntary MHCUs, as stipulated by the policy guidelines:

‘We don’t have the capacity, the nursing … stuff. We are very short staffed. For male ward which admits up to maximum of 50 patients, total nursing staff there [*it’s*] 13 nurses. So, it’s very difficult to … to allocate for shifts … The human resources is a problem …’ (NWP, FGD3, Participant B)

‘We not enough at all. Because remember the other wards, it’s mixed with a medical patient. It’s a capacity of 30 beds at a time, we will find 6 nurses on duty and then the other unit is 15 beds only mental 72 observation on a daily basis is 4, four or five staff members … So we are not the ratio is not enough.’ (GP, FGD2, Participant D)

‘We are struggling where it is not only that nurses and the doctors, even I have social things that I am struggling with … social worker, for the whole hospital for all the patients, so having to work on all the patients. It’s very difficult …’ (NCP, FGD1, Participant A)

**Sub-theme 2.10: Other resource-related challenges:** The MHCPs stated that they experience a burden from the challenges raised by the MHCUs who are unable to go home because of their low socio-economic status. After discharge, the MHCUs might stay longer than expected because of lack of money for transport to go home:

‘… I think we should also talk about other things … Now if you have a lab that is not up to standard, then we got a problem with retrieving the results. Sometimes they take long. Some of the results or investigations are not done in the house and they take longer than three days. And by then those users are supposed to be out of the hospital. It also creates a problem.’ (NWP, FGD3, Participant F)

‘So, in turn, so I’m thinking of enough sufficient availability of beds in placement centres, placements or places where people that have social problems can get that assistance, that we do have patients that have social needs that remain stuck in our wards …’ (GP, FGD2, Participant A)

**Sub-theme 2.11: Medical-legal risks:** Although the policy guidelines recommend a safe assessment environment for MHCUs, there are significant challenges in providing such an environment for 72-hour involuntary MHCUs because of infrastructural limitations. These individuals, who are often aggressive, uncooperative, and suicidal, require specialised care. When they are not admitted to a secure unit, their safety is seriously jeopardised, raising substantial concerns:

‘Patients have attempted. For example, suicide or the easy aggressive behaviour in the ward and things like that. But the ward is safe as far as I understand. Although patients are high risk or suicidal or abscond, we’d rather prefer them to go to tertiary institutions where they can be better managed.’ (GP, FGD2, Participant F)

‘Remember, the practice now is where our mental health users are being admitted amongst the other patients and those patients are so vulnerable and they are so helpless. In case there sedation didn’t go well or maybe yeah hazardous things normally happen to these patients.’ (NCP, FGD1, Participant C)

**Sub-theme 2.12: Patient care challenges:** The participants in this study disclosed that MHCUs either receive compromised care or are misdiagnosed, because of lack of relevant and competent MHCPs during their care, treatment and rehabilitation. The patient care challenge arises when the forms stipulated by the 72-hour policy guidelines are not completed correctly, having missed information and are not properly adhered to. The MHCUs cannot be managed properly because of missed history taking and this results in increasing re-admissions that cumulatively affect the finances of the hospital.

‘Oh with the finances, remember that if we are better taking care of the condition issue we will never spend a lot on those individuals … that patient will keep on coming to the hospital and may be every month or every second week, meaning that you waste, not really waste …’ (NCP, FGD1, Participant D)

‘I think the difficulty in real life is that the 72-hour process, as we’ve pointed out, that mental health care users often stay longer than. The stipulated time, according to the policy, so patients because it is a closed ward, patients don’t have recreational activities or access to the outside as as often as they would like. So that also causes a lot of frustration. It’s not necessarily the most suitable structure for a long-term stay.’ (GP, FGD2, Participant A)

**Sub-theme 2.13: Overcrowding in 72-hour assessment facilities:** The participants highlighted several challenges, such as a shortage of beds, insufficient infrastructure in the 72-hour assessment facilities and inadequate accommodation for MHCUs in psychiatric units. These problems contribute to overcrowding during the period of continued care, treatment and rehabilitation after their 72-hour assessment:

‘In terms of overcrowding, we might have more mental health care users than [*we can handle*]. A higher load than our team can comfortably manage, so we might have a [*large*] patient load.’ (GP, FGD2, Participant A)

‘To the extent that it should be, because of the overcrowding and everything else there, the infrastructure and everything is really not conducive to the users there.’ (NWP, FGD3, Participant C)

‘Yes, there is overcrowding.’ (NCP, FGD1, Participant B)

**Sub-theme 2.14: Readmissions and its consequences:** The MHCPs expressed concerns about the frequent readmission of MHCUs, noting that it contributes to overcrowding and the inefficient use of resources such as medication, further exacerbating the overcrowding issue in assessment facilities. This situation creates a significant challenge to the effective implementation of guidelines, as MHCUs often remain in the assessment unit longer than the prescribed 72-hour period:

‘With my discipline. If the users are kept here for a longer period and that’s quite challenging, challenging and like as these, what has been said as well that they go home because they are no longer psychotic and they end up reusing substance again and they come back into the system in again, and it’s quite a challenge.’ (NWP, FGD3, Participant C)

‘Remember especially that they can actually be brought back by the police. Already they come with transportation and so forth. They are not inducing only the financial concern from the hospital, but themselves and the socio economic of patients, family because they have to spend. Most of the time, you can’t put this patient in a taxi. You have to get a special transportation for them. It is a lot of money, and these people travel from far, or sometimes they end up using the police vehicles, of which they don’t have, so you are actually cutting a lot of costs. Both this side and also outside and where the patient resides as well.’ (NCP, FGD1, Participant D)

#### Theme 3: Mental Health Care Practitioners’ suggestions on strengthening the policy guidelines on 72-hour assessment of involuntary Mental Healthcare Users

The sub-themes that emerged from the MHCPs’ suggestions on strengthening the 72-hour policy guidelines on assessment of involuntary MHCUs are discussed hereunder.

**Sub-theme 3.1: Adequate infrastructure and resources:** The MHCPs suggest that it is important for 72-hour assessment facilities of involuntary MHCUs to have adequate infrastructure and resources. The 72-hour assessment facility as stipulated by the policy guidelines should be conducive, with adequate space to accommodate MHCUs, without compromising their safety, including service delivery. The following quotes emphasise these aspects:

‘We need the right infrastructure for the admission of the mental health users.’ (NCP, FGD1, Participant C)

‘We need support from Department of Health and with the infrastructure. To build appropriate infrastructures for the 72-hours so that we don’t mix patients.’ (NWP, FGD3, Participant B)

‘[*A*]nd even the infrastructure. It should be conducive for us to nurse mental health users.’ (GP, FGD2, Participant)

**Sub-theme 3.2: Accessible services (assessment, treatment and/or rehabilitation):** The MHCPs suggested to strengthen the implementation of policy guidelines on 72-hour assessment and to ensure that mental healthcare services should be available, accessible and affordable for all MHCUs at all times. The services should be available within MHCUs communities where rehabilitation centres must be available, and the mental health hospital should be close to their homes:

‘I think you … what we should … You know the best thing to do here is to strengthen or maybe … yeah, the district hospital [*must*] start observing their own patients. You know, they … we have to improve the infrastructure to cater for the 72-hour observation, at the district level, so that those users can be observed nearer to home, than to always to come to … to … to … come to …’ (NWP, FGD3, Participant F)

‘So, we don’t have Occupational Therapy some patients in the ward could benefit from OT, but we don’t have access to that. So like so for long term stay also not the, not the ideal situation.’ (GP, FGD2, Participant F)

**Sub-theme 3.3: Sufficient human resources:** Most MHCPs suggested more human resources must be recruited and retained for rendering care in the 72-hour assessment of involuntary MHCUs. Providing adequate human resources should be made a priority, and should incorporate not only skilled personnel but also security personnel and other stakeholders in order to ensure smooth facilitation of 72-hour assessment of involuntary mental health service provision:

‘Yeah, I think the infrastructure … it’s always part of our talking there. Because one security [*guard*] stands by the gate, whenever someone causes a problem we have to call him. The number should be increased.’ (NCP, FGD1, Participant E)

‘[*A*]nd understand staff too, the equipment processes. To improve the medical healthcare workers in the 72-hours their numbers are very short, they are very short staffed.’ (NWP, FGD3, Participant B)

**Sub-theme 3.4: Competent human resources:** The MHCPs suggested that provisioning of competent human resource in caring for the 72-hour assessment of involuntary MHCUs should be a priority. Involuntary MHCUs are unable to make decisions for themselves and, as a result, depend on MHCPs and other stakeholders to make decisions on their behalf. By ensuring the availability of skilled personnel, the implementation of the 72-hour assessment policy guidelines can be strengthened, leading to more positive outcomes:

‘I think it’s like participant F mentioned. Continuous training and workshops also then … distributing knowledge with regards how to complete the forms correctly. Thank you.’ (NWP, FGD3, Participant C).

‘I think, as we’ve mentioned, shortage of staff, we need more staff members and our staff members needs to be empowered. We need constant seminars regarding mental health, so that we can know how to implement these guidelines …’ (GP, FGD2, Participant D)

‘… But the in-service training needs to be done but sometimes I understand it some properly to be refreshed, so maybe it will depend on the institution we go with annually. If it need be.’ (NCP, FGD1, Participant C)

**Sub-theme 3.5: Governance of guidelines:** The participants suggested that all MHCPs and other stakeholders should be well-trained in and receive refresher courses regarding the 72-hour policy guidelines to ensure competency. This would assist in ensuring that the 72-hour policy guidelines are implemented to the letter, therefore preventing ill-treatment of MHCUs, and promoting good care towards the involuntary MHCUs:

‘So, so that is that also creates a problem and may be even … and so a more cohesive understanding throughout all levels of care and Health Administration. The policy guidelines would actually help to implement them better.’ (GP, FGD2, Participant A)

‘They should decide, there is quality also. Quality also assurance have to make sure that things are done. There must be monitoring and evaluation, of the implementation, it can’t just be left on its own.’ (NCP, FGD1, Participant B)

**Sub-theme 3.6: Community and stakeholder empowerment:** The MHCPs indicated the need for community members to be informed about mental illness through health education and community awareness campaigns. Such stakeholders would then have adequate insight regarding mental health. This should assist in reducing the stigma attached to mental illness and consideration towards the MHCUs could pan out positively:

‘I think as we need to do more of a mental health awareness to the community so that if they can also understand, they come on board. We need to do more of awareness in the communities. We should not look at the staff alone. We should also look at the community, do a lot of awareness for the community.’ (GP, FGD2, Participant D)

‘I think it will mainly have to be through community outreach.’ (NWP, FGD3, Participant E).

‘Through awareness campaigns.’ (NCP, FGD1, Participant E)

**Sub-theme 3.7: Family empowerment:** The MHCPs emphasised family members as crucial stakeholders when admitting and caring for the involuntary MHCUs. The reason being that they stay with the MHCU at home and have a full history of the mental illness of the MHCU, and the policy guidelines on 72-hour assessment of involuntary MHCUs prescribes their involvement:

‘I want to … add that we do when we have a family member that we do our best to educate and help the family member to … often they’ve got misconceptions and they’ve got prejudices … and we do our best to dispel those prejudices.’ (GP, FGD2, Participant A)

‘When they get admitted, normally one has to consent for them either close family member or whatsoever. So when they come because our people are not so well informed and there’s still stigma around mental healthcare users, they need to be well prepared and informed. What 72-hour observation is because most of them they just want to get to rid of their family members.’ (NWP, FGD3, Participant D)

‘Going back to that one of the family, that thing of counselling, they must also be taught about this thing of psyche to stop the stigmatisation, because sometimes they will be calling them crazy, so they must be taught that this thing happens. They must be taught about this *akere* [*isn’t it that*] we have different types of signs and behaviour or when you notice this you must see gore [*that*] it is this and this?’ (NCP, FGD1, Participant F)

## Discussion

The study’s findings will now be discussed in relation to the existing literature.

The policy guidelines on 72-hour assessment are in place to disseminate procedures for the facilitation of requisite care for MHCUs. These guidelines serve to ensure the effective management of required documentation related to 72-hour assessment of MHCUs. In addition, the MHCPs’ understanding regarding the policy guidelines is that it assures the MHCUs’ protection with consideration that the MHCUs can be verbally or physically aggressive. Stander, Hodkinson and Dippenaar (2021) attest that the involuntary MHCUs can be aggressive. In addition, Section 40 of Chapter 5 of the MHCA (Act No. 17 of 2002) permits the South African Police Services (SAPS) to oversee the MHCU’s welfare in a prehospital environment to ensure the safety of the MHCU and those around them. Moreover, the 72-hour assessment of involuntary MHCUs guides the MHCPs (professional nurses, medical doctors or psychiatrists, clinical psychologists or social workers) on procedures to be followed during assessment, care, treatment and rehabilitation of involuntary MHCUs. According to the MHCP’s understanding, during the 72-hour assessment, there is an opportunity to exclude the medical conditions during assessment. This finding is consistent with Chennapan et al. (2018) and MHCA (Act No. 17 of 2002), which support that during the 72-hour assessment of involuntary MHCUs, exclusion of medical condition, which may have resulted in the clinical manifestation of the disorder is required before the MHCU is admitted to a mental health institution.

Following approval for admission of the involuntary MHCUs, the MHCPs shared a concern regarding lack of 72-hour assessment facilities. In addition, existing facilities providing involuntary care, often seem inadequate to provide this type of service (Alabi 2022; Bergan 2024; Maila et al. 2020). Furthermore, Johnson et al. (2022) and Warburton et al. (2020) add that there is shortage of human resources, beds, insufficient community-based services, including stigma towards the MHCUs and their families, inadequate mental health emergency treatment services and rehabilitation services where involuntary MHCUs are admitted for assessment, treatment, care and rehabilitation. These factors contribute to poor quality of care provided to involuntary MHCUs (Müller et al. 2024). This culminates in continuous prolonged stay of involuntary MHCUs, possibly misdiagnosing of MHCUs, leading to increased numbers of revolving admissions and poor administration (Vanagundi et al. 2023). These findings are congruent to this study. The MHCPs were convinced that their lack of understanding regarding the application and interpretation of the guidelines is because of lack of full appreciation of the 72-hour policy guidelines (Potthoff et al. 2022).

The MHCPs advocate for accessible and affordable mental healthcare services for MHCUs. These findings are consistent with those of Ndetei, Mutiso and Osborn (2023), which support improving care towards the involuntary MHCUs. The recommendation is that there should be an integrated strategy to managing mental illness. In addition, Alabi (2022), Angiuli (2023), Wormdahl et al. (2022) and Herrman et al. (2022) support that the MHCPs need should to be prioritised through mental health workshops, trainings and being taken for academic qualifications to improve the quality and development of accessible services in a way that satisfies stakeholders’ demands and makes proper implementation of 72-hour policy guidelines of involuntary MHCUs possible. It has been documented that the 72-hour policy guidelines must prescribe sufficient and competent MHCPs that are needed to care for the involuntary MHCUs (Johnson et al. 2022; Newman & Kramer 2019; Matsea, Ryke & Weyers 2018; Myburgh 2022; Potthoff et al. 2022; Warburton et al. 2020). There must also be family involvement and community support (Herrman et al. 2022). This will promote family and community participation in awareness campaigns and other recovery-oriented practices when providing care for mental health and rehabilitation for MHCUs (Herrman et al. 2022; Ndetei et al. 2023). Furthermore, this could reduce the stigma attached to mental illness. According to Maila et al. (2020), this could reduce the moral implications of involuntary admission regarding legal restrictions as human rights measures are affected, and the MHCPs also attest to the disparities (Maila et al. 2020). Hasan et al. (2021) and Mahdanian et al. (2023) concur that from a human rights perspective, society – and policymakers in particular – must actively support the idea that every person needs protection regarding legal consideration related to their health needs to fully exercise their rights. However, a thorough mental health practice model that incorporates human rights into current mental health practices and services must be developed and implemented to improve service delivery towards the MHCUs (Mahdanian et al. 2023).

### Strengths and limitations

The following section discussed the strengths and limitations of the study. The contextual nature of the study, which required the researcher to collect data from the three provinces of SA (NWP, GP, and NCP), is one of the limitations. Electrical issues, particularly load shedding and irregular network availability, the FGD sessions had to be rescheduled multiple times, which extended the time needed to collect study data. It was also unfortunate that due to a variety of obligations, several facilities’ psychologists, social workers, and psychiatrists were unable to participate during data collection. Regardless of the limitations, MHCPs (professional nurses, physicians, psychologists, and social workers) who participate in the assessment, care, treatment, and rehabilitation of involuntary MHCUs for a period of 72 hours were included in the study’s data collection. The researcher managed to conduct the data successfully from the three provinces, with three themes derived. Although the results of the study cannot be generalise it can be applied to other setting with similar challenges.

### Recommendations

The study’s recommendations will now be highlighted in relation to the findings, focussing on the MHCPs’ understanding of the policy guidelines, the challenges they face during the implementation of the 72-hour assessment for involuntary MHCUs and the suggestions offered to improve the effectiveness of these policy guidelines.

There should be accessible mental health services during the provision of care to the involuntary MHCUs under the 72-hour assessment admission, treatment and rehabilitation. In addition, to ensure proper facilitation of the MHCA and the 72-hour policy guidelines, there must be trained human resource to ensure timeous delivery of documents between the 72-hour assessment, the MHRB members and the HHE. There must be other MHCPs who are adequately trained and have specialisation in psychiatry to ensure proper filling of forms, including proper assessment, admission and rehabilitation of MHCUs. To ensure good practice in the facilities, there should also be adequate infrastructure, community involvement, family and stakeholder involvement to improve mental health care. Furthermore, there should be amendment of the Act and regulations should be specific about the qualifications of MHCPs to ensure availability of competent MHCPs and other stakeholders. Lastly, research on how to improve adequate infrastructure, collaborative partnerships, administrative support in health setting must be performed.

## Conclusion

Mental Healthcare Practitioners are dissatisfied that the 72-hour policy guidelines are not properly implemented. For instance, lack of a secretariat causes delays in the distribution of documents. Mental Healthcare Users are not assessed in a safe environment because of improper infrastructure and being cared for by MHCPs who lack psychiatry-specific training or specialisation. There is also stigma attached to mental illness. However to improve care, there should be well trained administration personnel, qualified MHCPs, suitable infrastructure, community and family involvement. When providing care to involuntary MHCUs under the 72-hour assessment, there must be readily available mental health services. With proper implementation of the 72-hour policy guidelines, protection of MHCU rights could be attained and maintained, leading to the effective management of mental health illnesses.
